# Entropy? *Exercices de Style*

**DOI:** 10.3390/e21080742

**Published:** 2019-07-29

**Authors:** Rocco Gaudenzi

**Affiliations:** Max Planck Institute for the History of Science, Boltzmannstrasse 22, 14107 Berlin, Germany; rgaudenzi@mpiwg-berlin.mpg.de; Tel.: +49-(0)30-22-667-274

**Keywords:** entropy, conceptual analysis, history of physics, statistical mechanics, information theory

## Abstract

Since its inception, the concept of entropy has been known under a variety of guises and been used in an increasing number of contexts, achieving an almost rock star-like status in both the sciences and popular culture. The three most prominent “styles” which entropy has been (re)told in and which have determined its popularity are the thermodynamic, statistical and information-theoretic one, owing much to the work of Clausius, Boltzmann and Gibbs, and Shannon, respectively. In the relentless hunt for the core of the concept that spurred this development, connections with irreversibility and emergence of time, nature of probability and information emerged adding to its elusiveness as much as stimulating its proliferation and cross-contextual adoption. In this historical review, we retrace, through primary and secondary sources, the three main perspectives from which entropy has been regarded, emphasising the motivations behind each new version, their ramifications and the bridges that have been constructed to justify them. From this analysis of the foundations a number of characteristic traits of the concept emerge that underline its exceptionality as an engine of conceptual progress.


*A symbol is a reality that is greater than itself*
Pavel Florensky [1]

## 1. Introduction

What is entropy? If the questioner’s purpose were to suddenly embarrass a physicist for a moment, that enquire would certainly achieve its aim—not because there is no answer, but because there are too many: a simple selection from the known historical sources, limited for example to thermodynamics and statistical mechanics, presents us already with several different, seemingly unrelated and not unambiguous definitions. Together with its dual companion energy, entropy is one word among a range of more complex notions within the vocabulary of physics which has wildly and unceasingly continued to transcend its own boundaries—including the non-scientific ones. In the popular culture the concept evokes, as a quick survey allowed us to confirm, vivid pictures such as that of “a broken glass” and similar phenomenological renderings; it acts as a metaphor for “chaos”, “disorder”, “decay”, “heat”, or triggers (more or less) surprising associations like “a techno party”, “a consulting company”, down to the blunt “death”.

Within science and beyond the thermodynamic realm, entropy is found in a profusion of other contexts: computation, cosmology, mathematical physics, economics, complexity theory, evolutionary biology, to name here but a few. In history, the concept also features in the cosmogonic and religious debates that were animated by professionals and amateurs, as well as in those which interrogated fundamental issues on the role of probability and the nature of physical description.

But what has caused this cross-contextual adoption of the concept of entropy throughout history? Has there ever been one single “original” definition of entropy, or rather a patchwork of meanings? What is the historical thread that ties all these versions to the *ur*-entropy which emerged in the original context of thermodynamics (the prefix *ur-* denotes here, as is customary, the original, first form of the object it is combined with, which is supposed to embody its supposed basic or intrinsic qualities)? What has made entropy so appealing as to elicit its borrowing and penetration in many scientific and lay contexts, in the specialised fields and in the popular culture? In response to the latter question, one could argue that the reasons are various and entirely contingent: entropy has, case-by-case, stimulated a conceptual revision/reorganisation, aided the expression of a new concept; or was chosen, and continues to be chosen, as an allusive name for formally analogous concepts, simply because the richly polysemous, ready-made idea of entropy was preferred to the burden of coining a new word or expression. On the contrary, one could also argue that its fecundity is not coincidence but has a more fundamental grounding: the concept, born in and motivated by a specific physical context, nonetheless has captured a general underlying structure that transcends that original meaning and realm, and makes it therefore suitable for a large variety of other spheres.

Against the background of the questions posed, this review wishes to map out how and motivated by what the thermodynamic concept of entropy historically ramified into the two other parallel branches—the statistic and the informational—which in turn have formed the base of many of the different and manifold analogues that are presently in circulation. The aim of such an analysis is not to judge in hindsight how legitimate the conceptual ramifications were and are (for which authoritative literature exists), but rather to understand, at least partly, the reasons why the concept of entropy has stimulated the sustained conceptual progress that it did, in its own field as well as in a variety of other fields. With that in mind, we look over the historical development of the concept from its introduction as far as the early 1960s, emphasising at each turning point the aspects of it that, at least in the eyes of some, called for further progress or suggested the possibility of an extension of its meaning.

## 2. The Dawn of the Concept: Entropy in Thermodynamics Style

### 2.1. Clausius’ Coinage: Giving Names to Things

As is well-known, the concept of entropy makes its first appearance in the realm of thermodynamics. Expressing in words its mathematical definition, a typical textbook definition of entropy reads as follows:

(i) Entropy is function of the state of a physical system at *equilibrium* with all its parts whose variation is given by the ratio of the heat traversing the boundary of the system and the absolute temperature at which the infinitesimal energy transfer is carried out.

Certainly informative, such definition seemingly just renames the ratio between two primitive and measurable thermodynamic quantities (heat and temperature). It is only through some subtle considerations that one can reach a second definition which reveals the implicit consequences of the former and goes as:

(ii) Entropy [production] is the measure of the irreversibility of spontaneous physical transformations in an isolated system.

Since this alternative statement betrays the motivations behind its introduction of the concept of entropy and its core peculiarity, it is illustrative to historically retrace the pathway connecting the two.

The first definition tells us that, when an amount of heat, measurable as the sum of the work done on or by the system plus its change of internal energy ΔQ=W+ΔU, is transferred from a reservoir at an absolute temperature *T* to a well-defined system, the variation of the to-be-defined entropy of that system will be:

(1) $$\Delta S:=\frac{\Delta Q}{T}.$$

To ensure that at any moment the system is in thermal equilibrium, as required, one shall reduce the heat transfer to an infinitesimal amount dQ. This definition remains a rather useless statement unless ones notices, as Clausius did, that the quantity is endowed with the following particular key property. If our system is any mechanism which cyclically absorbs the amount of heat $dQ_{h}$ from a hot reservoir at temperature $T_{h}$ and subsequently dumps the amount $dQ_{c}$ to a colder one with temperature $T_{c}$ yielding mechanical work as the only result (such as the small displacement of a piston or a shaft against a resistance), then the following holds true:

(2) $$\frac{dQ_{h}}{T_{h}}=\frac{dQ_{c}}{T_{c}},$$

with the two members respectively defined as $dS_{res,h}$ and $dS_{res,c}$.

During the process, three well-defined physical systems are alternately involved: our machine, the hot reservoir and the cold reservoirs. (It is worth mentioning that in Clausius’ time the mechanism of reference was Watt’s steam engine, where the hot reservoir corresponds to the boiler and the cold to the water bath into which the condenser is immersed.) However, since at each and every cycle we observe the machine coming back to the exact same state (neglecting, of course, the infinitesimal wear and tear), its entropy variation $dS_{m}$ cannot be anything but zero, and thus only the two reservoirs will contribute to the total entropy variation dS. Using Equation (2), we arrive at:

(3) $$dS=dS_{m}+dS_{res,h}+dS_{res,c}=0.$$

That is, the *total* entropy *S* of the isolated complex machine + reservoirs is a conserved quantity. This allows us to construct the potential *S* in analogy with the energy potential, determined up to a constant and uniquely depending on two generic initial and final states *A* and *B*:

(4) $$S\left(A,B\right)=\int_{A}^{B}\frac{dQ}{T}.$$

Such is the state function arrived at and subsequently baptised “entropy” by Clausius in 1865 by composing two ancient Greek words with explicit parallel to “energy”. Since derived without reference to a particular system or transformation, this formula has a general validity. The fact, however, that we have made use of infinitesimal variations implicitly encodes a restriction: that each quantity macrosopically describing the state of the system shall take a well-defined value at any point along the transformation. Physically, this implies that the generic system that we have described should: (i) not experience friction; and (ii) transition between stationary states that are infinitesimally close to each other (by which it is said to be quasi-static or go “through states of equilibrium”). These are two conditions which make its transformations *reversible*. However, the thermodynamic systems in the real world are hardly ever of this type, thus one might wonder whether such an expression remains sensible if, say, we relax the second constraint. Let us see why it turns to be all the more so and what it tells us.

To break quasi-staticity. it is enough to suppose, for example, that the machine at *any given* time is appreciably colder (warmer) than the hot (cold) reservoir is put in contact with, so that the absorption (dumping) of a certain amount of heat occurs more quickly than the temperature equilibration. This translates into heat transferring between two bodies at different temperatures, which leads in turn to the uncompensated entropies:

(5) $$\frac{dQ_{h}}{T_{m,h}}=dS_{m,h}>dS_{res,h}=\frac{dQ_{h}}{T_{h}}\;\mathrm{and}\;\frac{dQ_{c}}{T_{m,c}}=dS_{m,c}<dS_{res,c}=\frac{dQ_{c}}{T_{c}}.$$

These uncompensated entropies break the inequalities in Equations (2) and (3) yielding instead for the total entropy variation always a value $dS^{non-eq}>dS=0$. Due to the presence of temperature gradients, to calculate the macroscopic total entropy variation one has now to compute one integral of the form in Equation (4) per each part of the system which has a definite temperature at each point of the transformation, and sum all of them. The total macroscopic variation so obtained will also *always* be larger than its reversible counterpart, so that this inequality can be used to demarcate and essentially characterise *any* non-quasistatic process in its most generic form.

The beauty of such an argument is that one can progressively do away with the machine—or, equivalently, take the limit for its efficiency going to zero, i.e., a mere conductive body—so as to effectively deal with a spontaneous evolution where no work is output. The result is a direct heat flow between the two reservoirs, with a consequent maximisation of the overall entropy production, the latter being larger the higher the temperature gradient. Since nothing in the process has been output to the outside world which could possibly be used to reverse the situation, that entropy production provides a measure of the irreversibility of the spontaneous process. Applying Definition (i) to real world systems, we now see how the Characterisation (ii) descends from it.

If we further suppose, as can be observed in the real world, that the reservoirs do not have infinite thermal capacity, we can also predict that within a limited time the system will reach thermal equilibrium, with entropy production coming to a halt and entropy locking in at a maximum value. The energy content of that final state is as high as that at the initial moment, but another *capacity*—which Clausius tentatively named “transformation content”, before settling on “entropy”—that allows the production of useful work has *dissipated* away. This evident and ubiquitous yet elusive capacity had certainly been expressed periphrastically by many of Clausius’ contemporaries (Thompson’s “principle of dissipation”, on which he insisted even after Clausius’ formulation, is an example), but it is only Clausius who gives it a *name* and a mathematically defined quantifying *symbol*. In so doing, after a long gestation (1854–1865), Clausius is able to restate the second law in an elegant and compact fashion in its volume on the mechanical theory of heat [2]:

If for the entire universe we conceive the same magnitude to be determined [..] which for a single body I have called entropy, and if at the same time we introduce the other and simpler conception of energy, we may express in the following manner the fundamental laws of the universe which correspond to the two fundamental theorems of the mechanics of heat.

1. The energy of the universe is constant
2. The entropy of the universe tends to a maximum.

Setting out to characterise and quantify reversible processes and the efficiency of heat engines, Clausius ends up with a measure for those that are irreversible, generalisable to no less than the universe at large, in this way transcending by far the original aim of the endeavour. The application of entropy to dissipation, irreversibility, or spontaneous processes can already be considered as being the first application of the concept of entropy outside of its original realm, and where the concept first shows its fecundity.

The truly *universal* character of the resulting formulation, as much as it might not surprise us nowadays, appeared to Clausius’ contemporaries, whether scientists or laymen, as an extremely powerful argument bounding the origin and final destiny of the universe. In the following, we give a flavour of the heated debate on cosmogony that ensued as an instance of the multi-fold repercussions of the concept of entropy.

### 2.2. Entropy in the Cosmogonic Context

As Kragh recounts in great detail [3], the discussion about the cosmological consequences of the by then embryonic second law of thermodynamics was initiated by Thompson and Helmholtz in the early 1850s—well before the introduction of entropy—in their more or less public addresses and popular lectures. Implying a statement on the beginning and the end of the universe, the arguments featured from the inception, and to some extent regardless of whether the proponent was in or outside the scientific community, almost inevitably a mixture of scientific and extra-scientific themes. These philosophical and theological overtones and the cultural resonance of the debate gave the lay person the opportunity and right to intervene, and the discussion continues in that spirit especially in extra-scientific circles.

In virtue of its prominent connection with the second law of thermodynamics and just a few years after its introduction, entropy enters the ongoing show in the form of the so-called “entropy creation argument”. The argument essentially states that, given the universe is not in equilibrium and its entropy always increases, then it must be of finite age and have an origin. The corollary that often accompanied this conclusion was that, if the universe had such a beginning, it must also have an end, which is to be identified with the heat death proposed by Helmholtz in 1854 in one of his addresses mentioned above. (It is important to remark that the presence of theological overtones did not prevent the scientific community from taking the argument seriously, especially when complemented with speculations on gravity, stellar evolution and differentiation of matter. As Kragh argued, George Lemaitre, the future proponent of today’s Big Bang, was likely influenced by such discussions.)

Benefiting from the inherent obscurity and ambiguity of its early days and entering the large cultural arena, entropy is popularised and detached from its scientific base. The association with chaos, disorder and decay that we observe in the general public’s understanding of entropy nowadays might owe to this early state of affairs. Incidentally, its equally general thermodynamic counterpart, energy, has had a similar destiny.

## 3. Entropy in Statistics Style

Returning to the more technical side of our story, it is useful to observe that, as early as 1857, Clausius had entertained and supported the vague ideas proposed at that time regarding a mechanical model of gas, and provided an extended version of such theories which included molecular rotational degrees of freedom. In the following year, in answer to an objection, he advanced the concept that we today call the “mean free path” [4]. The further consequences of that argument were drawn by Maxwell [5,6] and led him to propose that same year the well-known distribution that was given his name. Arguably, this is the first truly *probabilistic* law ever seen in physics.

Maxwell’s probabilistic thinking on the problems of thermodynamics drives him in 1867—stimulated by Tait’s request for feedback on a draft of his treaty on thermodynamics—to “pick a hole in the second law of thermodynamics, that if two things are in contact, the hotter cannot take heat from the colder without external agency” [7]; that is, to pick a hole in the second law as formulated by Clausius. The idea, elaborated on in the rest of the letter, is the demon’s thought experiment, according to which the “clever” action of “a finite being who knows the paths and velocities of all the particles by simple *inspection*” (author’s emphasis) can result into a transfer of the faster-than-average (slower-than-average) gas molecules contained in the cold (hot) vessel, to the hot (cold) vessel, with the net result of the hot gas getting hotter and the cold colder without additional work, thus decreasing the gas’ total entropy as the only net result. As Maxwell himself added shortly after, the chief purpose of the demon is to show that “the second law of Thermodynamics has only a statistical certainty”; and that if we are not able to play the demon it is because we are not “clever enough”. (Maxwell will restate these views at greater length providing several other examples and pictures, such as that of the bucket in the sea [8], and the equivalent of today’s reverse motion in a film [7].)

The introduction of these probabilistic aspects—the Maxwell distribution, the demon and the statistical certainty of the second law—as properties of the physical *description* rather than of the physics itself constitutes the beginning of the realisation of what a thermodynamic description is and to what degree it differentiates from the standard mechanical description. These same aspects can be also viewed in hindsight as having contributed to the opening of, respectively, two new routes in our entropy graph-tree: Boltzmann’s statistical reformulation of entropy, and the connection between entropy and information thanks to Szilard and Brillouin. Let us now concentrate on the former, leaving the latter turn for the next section.

### 3.1. Boltzmann’s Versions

Boltzmann’s interest in entropy and the second law of thermodynamics can be related, as Klein [9] argued, to his attempt to give a mechanical and atomistic demonstration of that law—the title of his 1872 issue “Analytic proof of the second law of thermodynamics” testifies to that. His endeavour can be characterised roughly into four steps, within which his views quite radically evolve and are accompanied by three distinct definitions of the entropy that gradually approach the well-known formula, which escorted him to the grave. In the following section, we briefly summarise and comment on the latter three (for the first two, we abridge the detailed treatment given in Klein [9]), emphasising at every step the change of interpretation that the concept of entropy underwent.

**1871** This first attempt is essentially devoted to spelling out the infinitesimal components of heat dQ and work dW when a gas is subjected to an infinitesimal change of internal energy $d\langle U\rangle$. The task is accomplished by expressing the latter two through the Maxwell–Boltzmann probability distribution and subtracting them to obtain an expression for dQ. After further manipulation, one can arrive at:

(6) $$S=\frac{2}{3}\log\int e^{\beta H(p,q)}d\omega +\mathrm{const},$$

where dω is the regular phase space volume element dpdq. Through this formula, Boltzmann first calculates the entropy of a monoatomic gas and then that of a system of independent oscillators, respectively, finding the first consistent with that derivable from standard thermodynamics, and using the second to work out the Dulong–Petit law for the heat capacity.

The essential and obvious difference between this expression and that of Clausius is that it features the microscopic molecular parameters, the positions and momenta of all the particles of the thermodynamic system, and uses them to compute a macroscopic property of that system.

**1872** The following year, Boltzmann [4] attacked the problem of entropy in a more general fashion, attempting to relate the macroscopic irreversibility to the microscopic evolution of the system and, with that, analytically *prove* the second law of thermodynamics. To do that, Boltzmann started from a probability distribution f(x,t) describing the number of (ideal gas) molecules possessing kinetic energy *x* at each time *t* and constructed its time evolution $\partial_{t}f$ “by considering how *f* changes during a small time interval as a result of collisions among molecules”. The details of this microscopical underpinning, contained in the partial differential equation $\partial_{t}f$, are not particularly relevant here. The quantity *H* is then introduced:

(7) $$H\left(t\right)=\int_{0}^{\infty }f\left(x,t\right)\log f\left(x,t\right)\;dx$$

and shown to *always*, i.e., for any $f\neq f_{\mathrm{Maxwell}}$, decrease in time until a stationary minimum is reached for which *f* is uniquely defined by the Maxwell distribution. Given the close relation between *H* and the thermodynamic entropy, Boltzmann concluded the result is “equivalent to a proof that entropy must always *increase* or remain constant, thus providing a microscopic interpretation of the second law of thermodynamics”. Worth emphasising is that the “always”, as mentioned in the expression, is to be taken literally: the law of entropy increase did not admit exceptions and was considered to be fully deterministic. (One might argue that, given the molecular chaos hypothesis implicitly used by Boltzmann to derive the time evolution of H(t), deterministic is not the right qualification for it. The sense given here to deterministic is that of non-probabilistic, necessary, and where no fluctuations from the predicted behaviour occur.)

This result, sometimes dubbed the “unrestricted” H theorem, attempts to give a mechanical grounding to that mysterious drift towards equilibrium and entropy maximisation that all systems seemed to exhibit, which had warranted the status of a principle. Looking at how this is practically reflected into the definition of entropy, we observe that the new ingredient is the dynamical description of the collisions (contained in $\partial_{t}f$), which imbues entropy with a certain time dependent aspect. This extends the microscopic definition in Equation (6) to *any* non-equilibrium situation allowing the tracing of the spontaneous tendency to equilibrium expressed by the second law. From an epistemological point of view, the attempt is to transform the law from a *principle* to the *demostrandum* of a theorem.

As much as that might seem a productive result, the strict determinism and the consequent *inevitability* of the time evolution were all based on an assumption that had sneaked in. Through what is today called the molecular chaos hypothesis—which allows the “forgetting” of the correlations existing between the particles after each collision—Boltzmann had inadvertently broken the Liouvillian evolution of the system and forced in the irreversibility. This realisation led to yet another radical change of perspective on entropy.

**1877** In response to a criticism in the form of the *inversion* paradox (for an integrated description of the inversion and recurrence paradoxes—the latter elaborated by Zermelo years later—see, among others, [10,11,12]) put forward by Loschmidt, Boltzmann was pushed to radically and critically revise some of the hypotheses at the basis of the evolution of the function H(t). The well-known Loschmidt argument claims that, if a given uniform distribution of gaseous molecules at time $t_{1}$ evolved from a non-uniform state—for example, a gas homogeneously distributed over a container and the same gas filling either side of it, respectively—then reversing the velocities of all the molecules shall lead back to the initial non-uniform distribution after the same time $t_{1}$. Therefore at least one configuration exists (that with all the velocities reversed in our case) which, chosen as an initial condition, would return an evolution dH(t)>0. This results in the paradoxical situation that the sign of entropy production, and with it the second law, depends *also* on the initial conditions, and is thus not universally deducible solely “from the nature of forces acting between particles”, as was claimed.

Recognising this exception to the previous strictly “deterministic” description, Boltzmann [13] developed a new argument according to which the microscopic reason for the second law stems from a point actually above and beyond the specific kinematics, and which rather lies in the sheer force of probability. This brief extract captures the essence of this shift:

Any individual uniform distribution, which might arise after a certain time from some particular initial state, is just as improbable as an individual non-uniform distribution; just as in the game of Lotto, any individual set of five numbers is as improbable as the set 1,2,3,4,5. It is only because there are many more uniform distributions than non-uniform ones that the distribution of states will become uniform in the course of time [4].

While each microscopic state (microstate, in short) taken *individually* is just as probable as any other, there are many more uniform states than non-uniform ones; as a result, the system *almost* certainly evolves into one of the former starting from a randomly-chosen initial state. While not denying exceptions, it just makes them highly unlikely on the basis of a *count* rather than of an intrinsic microscopic difference between an “ordered” and any other “disordered” state (a difference, for example, in their dynamics). Irreversibility is then not intrinsic to the dynamics—thus, as Boltzmann previously implicitly assumed, necessary—but a tautological consequence of probability: the measured macrostate is likely to be *that* one, because that is the most probable *relative to* the others. Let us see how this connection between entropy and probability is technically implemented.

Based on state counting, Boltzmann set out to calculate the relative probabilities of all the possible distributions f(x) in energy space—compatible with the particle number *N* and total energy *E* constraints, which is today’s microcanonical ensemble—and define thermal equilibrium, this time in statistical terms. Each distribution can be realised microscopically in a number of ways—called configurations, complexions, or permutations—given by the following equation:

(8) $$P_{f}=\frac{N!}{\prod_{n=0}^{p}f\left(x_{n}\right)!},$$

where $x_{n}=n\left(E/p\right)$, with n={0,…,p}, partitions the total energy in *p* cells. (Here, we have abridged the original argument by skipping the discretisation step adopted by Boltzmann, procedure which sparkled a large discussion, of which an exhaustive and up-to-date treatment is given in [14], initiated by Klein [15] and further discussed by Kuhn [16] in 1978 related to its supposed influence on Planck’s quantum.) Using Stirling’s approximation, one realises that:

(9) $$\int_{0}^{\infty }f\left(x\right)\log f\left(x\right)\;dx+\mathrm{const}=-\log P_{f}.$$

Namely, the integral previously associated with the negative of the entropy, *H*, results now to be proportional to the number of configurations in which a specific distribution can be instantiated. Evaluating the integral, Boltzmann shows that $P_{f}$ is maximised for $f=f_{\mathrm{Maxwell}}$ and yields, up to a constant, the entropy of an ideal gas in equilibrium as derived from standard thermodynamics. In principle, regardless of whether in equilibrium or not (this was Boltzmann’s conviction; in practice, these considerations apply at most to situations close to equilibrium), the entropy of the (measurable) macroscopic state of a system could then be: (i) at any moment given by its uniquely defining distribution f(x) (where no collision dynamics is now involved); and (ii) interpreted and computed as the number of permutations compatible with such distribution. In this framework, while the time evolution of f(x) towards the equilibrium is not strictly calculable, it is possible to compute the probability of the occurrence of a deviation from equilibrium to occur (a fluctuation). The second law can thus be reinterpreted as expressing a thermodynamic system’s spontaneous tendency to statistically move from less probable to more probable states and, reaching the most probable set (stationary equilibrium), constantly *fluctuate* around it. In the observer’s description, this translates as: the system *visibly* evolves until one specific stable and reproducible macrostate (that with the largest number of complexions) is found. after which the system stands still, its *macroscopical* evolution ceasing. This macro-, but not micro-physical, directed evolution and halting “explains” the flow of time and irreversibility as being a prerogative of thermodynamic systems, that is, for systems where a distinction of macro and micro is applicable. (For a critical review on the relation between thermodynamics, irreversibility and the arrow of time, see [17].)

Observing at a glance the path thus far, we have seen how the progressive addition of a microscopic perspective on entropy as a transformation content turns the second law from an apodictic and self-evident truth into the deterministic first, and then probabilistic, result of a theorem of evolution. The “explanation of irreversibility” that Boltzmann originally sought to give by relating entropy to the dynamics of microscopic states failed: its microscopic interpretation notwithstanding, entropy remains a function of a thermodynamic state, of an ensemble of microstates rather than of one single state; and *exists* only in the thermodynamic description.

The value of Boltzmann’s probabilistic reformulation of entropy is not diminished in the face of its serendipity. Despite Clausius’ entropy not being a “mechanical” variable (such as pressure, temperature, magnetisation, and so on) but an extensive potential, *through* probabilistic considerations it acquires a microscopic meaning and way of being computed. This constructs the bridge between the fundamental thermodynamic potentials and the mechanical variables which is at the core of statistical mechanics, thus practically allowing the construction of the state equation of a large number of systems from microscopic combinatorial considerations.

The probabilistic nature of the formulation of entropy proposed by Boltzmann also impacts on the entropy creation argument presented in Section 2.2. Some of the scientists who had adopted the deterministic view promoted by the H theorem, successively retreated from it on the basis that a law which allows exceptions in time cannot warrant general conclusions on the universe as a whole and for all of eternity. Boltzmann himself engaged in the discussion at the 1894 meeting of the British association at Oxford, and in the following year proposed an elegant solution [18]: an infinite universe in equilibrium might statistically contain enough “worlds” (i.e., planetary systems or analogues) where in at least some of them arbitrarily large deviations from equilibrium may occur; one of them may well be the world we live in, a pocket of local order allowed by one such large and long-lasting fluctuation.

From a broader viewpoint, Boltzmann’s reformulation contains in nuce its own generalisations: it allows the use of the concept in every context in which a *relative count* is involved; it is the foundation for the extension of entropy to quantum mechanics; and it provides the blueprint of a framework unifying entropy, irreversibility and disorder. The American physicist Josiah Willard Gibbs, partly independently, is also responsible for some of these important developments. In the following, we sketch how Gibbs prophetically captures essential aspects of the aforementioned blueprint starting from a different viewpoint, intuiting key elements that were not clearly explicated by Boltzmann and would turn out to anticipate subsequent interpretations of entropy.

### 3.2. Gibbs’ Version

In the chemistry-oriented 1878 long communication “On the equilibrium of heterogeneous substances”, Gibbs set out to apply the concepts of Clausius’ thermodynamics to the mixing of substances. In one of the sections of the long communication (as remarked on by Jaynes [19], the section in question was largely forgotten due to a mix of the idea being too ahead of its time and the obscurity of the language in some of its passages), he focused on the increase of entropy due to two gases, equal amounts of which are initially enclosed in each compartment V/2 of a container at the same temperature and pressure, and that mix by diffusion as soon as a partition is opened. Exclusively on the basis of Clausius’ entropy and Maxwell’s thermodynamic potentials, the entropy increase in the process is found to be (with *n* being the number of moles and *R* the ideal gas constant):

(10) $$\Delta S:=S_{final}-S_{initial}=2nR\log V-2\left(nR\log V/2\right)=nR\log2,$$

expressing the fact that a positive entropy increase occurs as a consequence of the mixing. Is this result independent of the nature of the gases? The answer is, Gibbs argued, “to some extent”, since an important distinction needs to be drawn.

On the one hand, when the two gases are assumed to be distinguishable, the result is consistent with the fact that an external work TΔS would need to be done on the system in order to compress back the two gases into their respective halves, when displacing for example a semi-permeable membrane. On the other hand, this is inapplicable when the two gases are composed of molecules that are *not* distinguishable (in which case, correspondingly, the displacement of the membrane requires no work, i.e., the TΔS is actually zero): its application would otherwise lead to the paradoxical situation that initial and final states are thermodynamically equivalent—since it does not make a difference whether the partition is there or not in this case—and yet different in terms of entropy; or, equivalently, that the entropy can be decreased (and increased) at no energy expense, in violation of the second law of thermodynamics. Spurred by this apparent inconsistency in the definition of entropy, Gibbs is pushed to reflect upon it and complete his more operative definition of the concept.

In the case of indistinguishable particles, when we affirm that the “energy and the entropy of the gas masses when mixed are the same as when they were unmixed”, it is because “*we do not recognise any difference* in the substance of the two masses” (author’s emphasis). Applying the same rationale to the case of distinguishable particles, when we say that the external work TΔS is needed to bring the system back to its original state, by the qualification “original” in fact we mean only “indistinguishable from the previous one in its sensible properties”, and not indistinguishable in the exact location of all its particles. (For the same reasons of avoiding ambiguity, above we say “compress back the two gases into their respective halves”, rather than “restore all the molecules in their previous state”.) Only with respect to such macroscopically stable and incompletely defined states—of which the thermodynamic description consists—the problems of thermodynamics are formulated.

From such operational elucidations, it follows that if we could conceive two gases that are arbitrarily similar for all practical purposes—for instance differing only in sensible properties which we cannot measure, in other molecular properties we do not (yet) know of or that do not enter the thermodynamic description—their mixing entropy would be independent of the degree of (dis)similarity between them. Thus, as much as the entropy of mixing is independent of the nature of the gas—the only assumption behind (10) is the ideal gas—its value must depend, in a discontinuous way, on whether we are able to distinguish or resolve the difference between the gases on the two sides of our container. On the same line, one might argue too, Gibbs suggested, that the entropy resulting from the mixing between distinguishable gases would differ from that between two non-distinguishable gases even if the process is characterised by the exact same microscopic dynamics, absolutely identical to the last detail, simply because we can restore the former to their original state, while for the latter this is entirely impossible.

Through this thought experiment, Gibbs concluded, as did Boltzmann, that entropy is a quantity that cannot depend on the (intrinsic) particle dynamics, but that it rather depends on our ability to recognise the differences and practically use them to act on the particles. (To remain not too abstract, such recognising of the molecules and “acting” on them is for example implemented by means of the regular semi-permeable filter we mention above: a membrane porous to all molecular species but to one, which allows both *extracting* work from the mixing process and using it to *compress* back the molecules into their respective compartments, at no net entropy expense. In this operational sense, extractable work, and thus entropy, depend on the technological capabilities available.) Failing to recognise these aspects, in which “entropy stands strongly contrasted with energy” as Gibbs stated, leads to the paradox we laid out at the beginning of the section.

It is evident how some of these conclusions resonate with the Maxwell demon’s arguments and were also voiced, more implicitly, by Boltzmann. What is striking however is that Gibbs arrived at them by following a different path, starting directly from the thermodynamic entropy and featuring a mixture of technical and conceptual-definitional analyses; and surpasses both Maxwell and Boltzmann in prophetically anticipating how epistemic aspects such as experimental resolution and lack of information affect entropy’s very core (incidentally, as Jaynes [19] noted, in Gibbs’s later work (1902) [20] an embryonic form of the principle of maximum entropy we encounter below also appears).

## 4. Entropy in Informational Style

In the previous section, we elaborate on the consequences that the probabilistic shift had for entropy and the second law of thermodynamics. We now see a further conceptual branching that opens a pathway which links entropy to information more concretely, explicating and generalising some of Boltzmann’s and Gibbs’ stubs. This time, the casus belli is the poking of the annoying Maxwell’s demon, which had remained subdued for quite a while. (For a comprehensive and detailed treatment of the repeated revivals of the Maxwell demon, including the original sources, see [21,22].)

### 4.1. Resurrecting Maxwell Demon

In a well-known 1929 paper, Szilard [23,24] proposed a deconstructed version of the demon thought experiment, which consists of a cylindrical chamber, in contact with a heat bath at temperature *T* and containing a single molecule. In contrast to Maxwell demon, the action that this demon can perform on the chamber is operationally limited to: (i) the insertion of a diaphragm-piston, which splits a chamber into two equal volumes V/2; (ii) the detection of the binary “presence/non-presence” of the molecule in one of the newly formed chambers; and (iii) the switching of a lever up or down, as a consequence of the detection, which determines the direction of the motion of the piston and the consequent expansion of the single-molecule “gas” back to its initial volume *V*. This setting allowed Szilard to strip the demon of its confounding properties, recognising that:

1. The result of the detection has to be physically instantiated in the position of the lever. The measurement process occurs only with some kind of temporary *memorisation*.
2. The entropy gained, as a consequence of the detection, in the isothermal expansion V/2→V, i.e., ΔS=klog2, has to equal the entropy cost of storing the binary information S=klog2.

The seemingly extra-physical and abstract cognitive actions of the Maxwell demon—involving the discerning capabilities of a living being and miraculous hands—could now be resolved operationally and simply quantified in terms of the stored information. In turn, information could be correlated with a physical process and treated as such. Restoring the validity of the second law of thermodynamics was accomplished by enlarging the physical system so that the formerly extra-physical processes could be included within the physical picture. By doing so, entropy virtually extended to all the physical processes involving information exchange, not only just to thermodynamic (Clausius), electromagnetic (Planck) and mechanical (Boltzmann) phenomena.

Szilard’s thought experiment—analogous versions of which have since become a reality in physics laboratories, where the single molecule of gas has been alternatively represented by a colloidal particle [25], a single electron [26], a microscopic bead [27], or the quantum spin of a molecular nanomagnet [28]—can be viewed as another bifurcation in the way out of the original concept of entropy: One the one hand, it became the starting point of Brillouin’s extended framework generally linking physical entropy and information [29,30], and was followed by the Landauer principle [31] (we refer the reader to [32] for a review of this thread). On the other hand, it spurred the enquiry into the relation between our “subjective” (epistemic) state of knowledge of a system and its entropy, which was tacit in Boltzmann’s view and expressed in general terms by Gibbs. Although the two threads are intertwined, following primarily the latter thread gives us the chance to explore yet more radical reformulations of entropy.

### 4.2. Literal Interpretation of Szilard Entropy Cost of Information

Pushing the interpretation of the Szilard article further, one can conclude that the knowledge of the position of a particle is in fact *already* work: a sort of “epistemological” work which can be converted at wish into an *actual* work. (It is worth noting that, by making the extractable work depend on the information we have on the two molecular species, Gibbs’ treatment implicitly contains this argument.) Von Neumann proposed this idea when he stated, a few years later in 1932, the following (author’s emphasis):

[By letting the gas expand in the Szilard experiment,] we have exchanged our knowledge for the entropy decrease of klog2. That is: in volume *V* *the entropy is the same as* that in volume V/2 under the assumption one knows in which half of the container the molecule is located.

Independently of whether the partition is physically inserted or not, i.e., whether the phase space of the system has been halved or not, the entropy decrease occurs as soon as the position of the particle is known. Reciprocally, if the partition is inserted without knowing where the particle is physically located, no useful work is attainable. In other words, for as long as the measurement is considered valid and regardless of the physical presence of the partition, the state of knowledge *sets* the entropy of the system: logical information *on* the system and entropy *of* the system are equivalently *physical*. Is then the entropy, at least at the instant of the measurement, different for the subject that has measured it than for someone who has not measured it? In other words, is entropy in the mind of the beholder?

Two decades later, a philosophical debate began among, among others, von Neumann, Pauli and Carnap, on the nature of entropy and information as thermodynamic, logical and information-theoretic concepts; how they are to be interpreted and whether they should be distinguished or can be conflated. Referring to Köhler [33] for a thorough analysis of the argumentations, we now move to Shannon’s information-theoretic entropy.

### 4.3. Entropy According to Shannon

In 1948, quite independently from speculations on the Maxwell demon and in the engineering context of Bell Labs, Shannon published an important paper on a mathematical theory of communication [34]. What is particularly relevant for this exposition is his purpose of extending the existing theory (advanced by Nyquist and Hartley) to the savings that can be obtained “as a result of the statistical structure of the original message” (and “the nature of the final destination of the information”), by which he essentially meant to characterise the compressibility limits of any *general* message given the statistical knowledge about the source. At the core of such an extension lies the concept of entropy, although interpreted quite differently here, as described in the following.

Deconstructing communication, one arrives at three essential components: an information source at one end of a (say, telegraph) line, the line itself and a receiver at the other end. In terms of these components, the whole problem of communication, Shannon maintained, is reduced to the receiver being able to make up the data sent by the source based on the physical signals received. The information source can be thought of as a system which “draws” a random letter *x* from an n-letter alphabet $x_{1},\ldots ,x_{n}$ with probabilities $\left\{p_{1},\ldots ,p_{n}\right\}$ at each time sequence $t_{i}$. The goal is to find a measure $H\left(x\right):=H\left(p_{1},\ldots ,p_{n}\right)$ of how uncertain we are about the outcome of the event. Imposing few basic general requirements about the characteristics of such a measure, it is shown that:

(11) $$H\left(x\right)=-K\sum_{i=1}^{n}p_{i}\log p_{i}$$

is the only possible alternative. Among the properties one finds inspecting *H*, a few are of particular interest in order to gain an intuitive understanding of its meaning:

1. *H* takes the maximum value $\left(:=H_{max}\right)$ when the states *i* are equiprobable—and monotonically increases ∝logn with the number of states—and conversely is minimised by a probability distribution of the type {1,0…,0}, where a single choice is available.
2. Given two events *x* and *y* with some degree of mutual correlation, the function *H* of the joint event H(x,y)≤H(x)+H(y), where the equality holds only if the events are uncorrelated.
3. Any averaging operation on the $p_{i}$, i.e., an operation whose result equalises the probabilities in any degree, corresponds to an increase of *H*.
4. The probability distribution which maximises the functional *H*, on the only additional condition that a standard deviation for *x* is fixed, is a Gaussian distribution.

An example of the application of the first property is illustrative to visualise how *H* is meant to be interpreted. If our distribution is peaked on a single value, at each turn, the letter that the source produces is certain: performing the draw does not return any new information, and the receiver will be able to predict/reconstruct the (trivial) message resulting from the sequence with a however noisy or even interrupted line, for effectively the message has zero information *content*. As opposed to it, if each letter is equally probable, the sequence will be as unpredictable as possible and its information content maximal. The receiver of such a message will not be able to reconstruct it unless they listen through a lossless channel, for every single signal—be it a telegraph pulse or a bit—is as essential as any other to the “content”. In practice, this translates into the fact that the latter case there is no room for compression—regardless of the encoding, the number of bits remains the same and maximal—and, conversely, that any redundancy and/or correlation, e.g., between successive letters in the words of a specific language, renders the draw more predictable and, upon a “smart” encoding, allow for compression. This view suggests that, in the information-theoretic framework, information, uncertainty, ambiguity and freedom of “choice involved in the selection of the event” are synonyms (inversely proportional to predictability and compressibility) and all possible names for *H*.

As one might remember, Boltzmann entropy deals with a situation that is partially analogous to that just presented. In the expression of the entropy, $S=k_{B}\log P_{f}$, the argument of the logarithm represents the number of (unobservable) microscopic configurations compatible with an observable macrostate *f*. These configurations ($n=P_{f}$) were supposed as all being visited with equal probabilities $1/P_{f}$, which, inserted as $p_{i}$ in Equation (11), returns precisely Boltzmann entropy. Conversely, a perfectly ordered physical configuration (for which $P_{f}=1$) has, in analogy with the sequence of identical letters, zero entropy for there is no ambiguity about the microstate given the observed macrostate.

As much as the parallel between Shannon and Boltzmann might seem at first surprising, a reinterpretation of Boltzmann’s formula from an operational and “subjective” perspective provides the hint and dispels the surprise: if the observer (or experimenter) were similar to Shannon’s receiver, and each probing of a microscopic state from the set of allowed states were seen as a draw from a pool of possible letters, the observer’s uncertainty about the microscopic state should be essentially the same as the one about each letter in the textual sequence. The observer is ignorant about the experimental system in the same measure in which a receiver is before her information source.

While it is hard to say whether Shannon meant, had thought of or entertained to any extent this interpretation, what we know is that he decided to call its functional *H* “entropy” and was made aware of the formal analogy between the two expressions—which becomes an equality if Gibbs entropy, derived from Boltzmann, $S=k_{B}\sum_{i=1}^{n}p_{i}\log p_{i}$ is taken—as recounted in this famous anecdote (reported in [35]):

[When searching for a name for *H*] I thought of calling it “information”, but the word was overly used, so I decided to call it “uncertainty”. [...] von Neumann told me, “You should call it entropy, for two reasons. In the first place your uncertainty function has been used in statistical mechanics under that name, so it already has a name. In the second place, and more importantly, nobody knows what entropy really is, so in a debate you will always have the advantage.”

As is clear from the passage and in accordance with the views laid out in Section 4.2, if not Shannon, von Neumann was inclined to regard the formal similarity between thermodynamic and information-theoretic entropy as a pointer to an underlying identity. In choosing a name for the symbol *H*, while Shannon might have wanted to vaguely recall some aspects of the concept of entropy and at the same time extend its accepted meanings, von Neumann’s preference for an already strongly characterised concept wishes to make the statement that the probabilities $p_{i}$ appearing in Boltzmann and Gibbs entropies are to be interpreted as reflecting the lack of knowledge of the observer concerning the physical system, with an increase of its entropy as reflecting a corresponding increase of the uncertainty of the observer about the microscopic situation. (There is some controversy with regard to von Neumann’s suggestion and its implications: for example, Tribus and McIrvine [35] saw that behind von Neumann’s irony lies his serious conviction that here was an opportunity to finally clarify Clausius’ vague concept, while Denbigh [36] argued that von Neumann’s decision did science a “disservice”.)

Quite independently of the repercussions on physical entropy, as we show in Section 4.5 and Section 5, Shannon entropy and the related information would thrive in their own domain and inspire numerous analogous concepts in other fields.

### 4.4. Information-Theoretic Interpretation of Physical Entropy

While von Neumann certainly considered the formal analogy not to be coincidental and connected it to the role of the observer, one had to wait another decade to see a radical and more general reinterpretation of statistical-thermodynamic entropy along the “subjective” line we sketched and detached from the material side of information expounded in Section 4.1. The development of this perspective, with its technical implications, is thanks to Jaynes [37], who set the stage with these words:

The mere fact that the same mathematical expression occurs both in statistical mechanics and in information theory does not in itself establish a connection between the two fields. This can be done by finding new viewpoints from which [the two] entropies appear as the same *concept*.

The program laid out in this paragraph essentially proposes the reversal of the line of reasoning which had been used until then to *arrive at* the concept of entropy, and instead takes Shannon entropy as the starting concept. The rationale behind it and the reason one can do so, Jaynes maintained, is the fact that Shannon entropy “has a deeper meaning, quite independent of thermodynamics”. Let us briefly sketch out what it is meant by this and its implications for the concept of entropy.

In statistical mechanics, the description of a thermodynamic function, for example, the total energy, starts from the equation of motion $H=H(x_{1},\ldots ,x_{j},\ldots )$. Assuming that each $x_{j}$ can take the discrete values $x_{i}$ (i=1,…,n), one can write:

(12) $$\langle E\rangle =\sum_{i,j}p_{i}\left(x_{j}\right)\;H\left(x_{1},\ldots ,x_{j}=x_{i},\ldots \right).$$

To move any further and calculate the other thermodynamic variables from the same microscopic variables, if the average value is all that is known—as is typically the case—one is forced to make a hypothesis on the probabilities $p_{i}$. One such case, it might now be realised, was Boltzmann’s assumption that all the microscopic configurations are equiprobable (Gibbs ergodicity is another example of such a hypothesis). All there is to justify that hypothesis is that a priori there is no sufficient reason to choose otherwise, that is, the evoking of Laplace’s principle of insufficient reason. However, is there a way, Jaynes asked, to instead rigorously account for that choice? Similar to what happens in applied statistics, the problem is to find a probability assignment which reflects the information at disposal, without introducing additional biases. The answer is found when turning to Shannon entropy: the correct probability distribution is the one that, plugged into Equation (11), maximises it subject to *our* knowledge of the system (average value(s), and/or bounds on these values).

With this, more Bayesian, principle of maximum entropy at its core rather than the (frequentist) one of insufficient reason, statistical mechanics becomes the science of making constrained inferences *about* a physical system based on the partial information (uncertainty) that the experimenter has of it, and where entropy, as a primitive concept, supplies the criterion to quantify and incorporate this (lack of) information. In this view, thermodynamics and the thermodynamic entropy are but the results of that branch of a general theory of statistical inference which deals with physics’ experimental situations. (For an exhaustive treatment of the numerous applications of the principle of maximum entropy, see [38]. We note in passing that this view has inspired other programs of establishing physical theories as necessarily descending from similarly general principles, see, e.g., [39].)

Insisting on this Bayesian interpretation, Jaynes [40] further stated that entropy, besides measuring the ignorance/capabilities of the observer, is an *anthropomorphic* concept even when considered at the phenomenological level. (For a further elaboration of these views, see Toffoli [41] and references therein.) Since physical entropy always presupposes a set of parameters defining what for us is the complete thermodynamic state, it is in fact a property of a *description* rather than being an intrinsic property of a system. “True”, measured entropy variations indicated in engineering steam tables do not, for instance, include electric field strength as a parameter, a parameter which would affect the entropy significantly, given that water molecules have a strong electric dipole. Similarly, as Jaynes argues in the context of the Gibbs’ paradox [19], if in a description we do not specify the number of molecules—or equivalently do not know whether their number may change—we might be observing a variation of entropy that is incompatible with the second law. In line with Gibbs, Jaynes regarded entropy as depending on our ability to detect differences in experimental situations.

In both of the above senses—in their reflecting uncertainty and being description-dependent— physical and information-theoretic entropy are, according to Jaynes, “the same concept”.

### 4.5. The Concept of Relative Entropy

Jaynes’ view on entropy, conjoining Gibbs’ early intuitions and Shannon entropy, suggests treating de facto thermodynamic experiments as statistical “surveys”; in other words, problems of thermodynamics are statistical problems coping with an intrinsically limited knowledge and guided by the question of how much one can infer about a sampled population from a certain set of observations. In this view, the experimenter designs and conducts an experiment seeking information about the system under investigation, just as a statistician would do. The problem of how to assign a probability distribution constrained by the experimenter’s state of knowledge thus becomes relevant and, as we have seen, is resolved (partially) by the principle of maximum entropy. However, in addition to the constraints—and this is more directly relevant to statistical inference, than to physics—a prior distribution might also need to be factored in, which poses then the question of to integrate that into the picture. In the context of attempts at generalising information-theoretic entropy to the abstract field of statistical inference in the early 1950s, the concept of relative entropy emerges to address this demand. It is informative to briefly review it to see how Shannon entropy (and Jaynes’ view) have inspired a revision of the concepts of information and information measure in contexts more abstract than communication engineering.

In 1951, Kullback and Leibler [42,43] proposed giving a definition of distance, or divergence, between any two statistical populations “in terms of *our* measure of information”, that is, a measure closely connected to Shannon entropy rather than to previous definitions of information used thus far in statistics (they referred in particular to Fisher information [44] defined as the amount of information that is contained in a random variable *x* about an unknown parameter θ of a certain model f(x,θ)). The principle behind the divergence, they maintained, shall be that of the statistician, who estimates the difference between a given probability distribution f(x) and a reference distribution $f_{r}\left(x\right)$ “according to how difficult it is to discriminate between them with the best test”. With these ideas in mind, they proposed “the mean information for discrimination”, alternatively called divergence or relative entropy, to be:

(13) $$I(f||f_{r})=\int f\left(x\right)\log\frac{f(x)}{f_{r}\left(x\right)}\;dx.$$

This expression, which bears close resemblance to Shannon entropy, has interesting interpretations and uses. In its first appearance, the logarithm of the likelihood ratio $f\left(x\right)/f_{r}\left(x\right)$ is seen as the weight of evidence in favour of the hypothesis *H* (the variable *x* is described by f(x)) against $H_{r}$ (the variable *x* is described by $f_{r}\left(x\right)$) given a specific instance of *x*. Regarding $f_{r}\left(x\right)$ as a prior, the maximally non-committal among the distributions satisfying the given constrains is the f(x) which minimises the relative entropy. This is the so-called minimum relative entropy principle, which generalises Jaynes’ maximum principle to the inclusion of a prior and reduces to it when the prior is a uniform distribution [45,46]. In the Bayesian framework, relative entropy represents the information gain obtained revising a prior reference distribution to the distribution *f* as a consequence of a new fact. When referred to modelling, it is interpreted as the amount of information lost when approximating the “true” probability distribution *f* with a model (or any description) $f_{r}$. In a variety of contexts related to applied statistics, it is used to measure evidence or “surprise”. (For an early example of this interpretation, see [47].)

## 5. Other Fruits of the Entropy Tree

In the preceding sections, we target the major paradigm shifts, guided by formal or interpretive links, that the concept of entropy has gone through, only mentioning the “applications” of each paradigm summarily—the quotations marks are used because it is not always easy to establish the boundary between an innovative application and a conceptually new version. Here, we briefly expand on three relatively early cases and illustrate how in fact the creative exercise of “finding”—discovering or inventing, depending on the credo—such applications took place and what any common traits between them might be.

**Entropy and Cosmology** As we shown above, the entropy creation argument shaped the cosmogonic and cosmological views of the late eighteenth and early nineteenth century. Some hundred years later, entropy went into the universe once again, this time in connection with the most mysterious large scale objects in the cosmos, and which were thought to be beyond thermodynamics. In the 1973 paper “Black Holes and Entropy” [48], Bekenstein drew our attention to a series of enticing analogies between black-hole physics and thermodynamics, and in particular Hawking’s proof [49] that the surface area of the black-hole event horizon can never decrease. Based on this analogy and other thermodynamic considerations, Bekenstein arrived at a formula $S_{bh}\propto A$ linearly relating the black-hole entropy to the area of its horizon. The starting point is the relation between information and entropy as interpreted by Szilard, von Neumann and Brillouin, according to which entropy measures the uncertainty about the actual internal configuration of the system; and any new relevant information acquired (lost) acts to reduce (increase) its entropy. Considering then that, as Bekenstein maintained, anything falling into the black hole is rendered truly inaccessible to the external observer, including the information about its internal state, the entropy of the black hole should increase at least by an amount equal the entropy of the falling body.

**Entropy and Economics** As one of the stems of Shannon entropy, the first quantitative applications to economics—two examples being demand analysis and economic equality—can be noted in econometrician Henri Theil’s work in 1965 [50,51]. Interpreting the $p_{i}=x_{i}/N\bar{x}$ appearing in Equation (11) as the income $x_{i}$ of the individual *i* in a set of *N* people divided by the total income $N\bar{x}$, Theil’s entropy $S_{Th}$ gives a measure of economic equality. From this, one can define, in complete analogy with Shannon’s redundancy, a differential $T=S_{max}-S_{Th}$ between maximum equality ($p_{i}=\bar{x}/N\bar{x}$) and actual equality. In an economics context, this index can be used to measure inequality, wealth isolation or segregation; in evolutionary biology and ecology, it is an index of biodiversity. In the same vein, the properties shown in Section 4.3 and elsewhere demonstrate the way Shannon entropy is suitable to quantify variety, richness, diversity.

In close conceptual analogy with the thermodynamics of irreversible processes, in 1971, the economist Georgescu-Roegen [52] proposed viewing human economy as a system that transforms matter and energy from valuable resources that humans can use into high-entropy non-valuable waste and pollution (in fact, at different scales, the Earth’s biosphere, the human economy and the living organisms are nested dissipative systems out of equilibrium), resulting in increasing “entropy” of the system nature + economy. Considered today seminal in ecological economics, the concept of entropy served to introduce in economics the notion of limitedness and irreversible degradation of natural resources caused by economic activity into economics. This doctrine, and its suggestion of inevitable consequences for the future—a form of heat death version restricted to the planet Earth—would be later dubbed “entropy pessimism”. Entropy once again is made a messenger of death.

In each of the three presented situations, a different version of entropy is chosen as a starting point, and some form of analogical link is established between that version and a phenomenon or a quantity in the domain to be modelled, in this way extending and/or generalising entropy’s domain of application. In the first instance, the analogy is drawn between two physical systems whose respective characteristic quantities prove to share a common quantitative behaviour, which constitutes the starting point for the extension of entropy’s domain to another class of physical objects. In the third case, a more qualitative analogy is established between two distinct realms which show common phenomenological aspect: a thermodynamic system with respect to its surrounding on the one hand, and the human economic system with respect to the natural environment on the other hand. (Here the analogising of such aspects and the concentration on the dark consequences remind us of the popular readings.) In the second application, the inequality index, the analogy is material (between abstract objects) and allows the recognition in the information-theoretic entropy of a measure of how distributed/spread a pool of “objects” might be with respect to a certain generic property (not only their occurrence probability)—this is done by replacing the set of probabilities in the formula with “frequencies” more generally intended. This last case is the paradigm of how the generalisation of the concept of entropy has occurred in many other situations and the term entered a variety of contexts. All three cases show in different ways how entropy has transcended its original respective domains of application.

## 6. Summary and Conclusions

Entropy is a concept of humble origins and modest pretensions: it was marked by and aimed at the problems of bulky and mundane machines; meant to function in dusty factories, rather than in laboratories; addressed to the physics at the human-scale, far from the fancies of the cosmic stage, the micro-world, and the philosophers’ drawing rooms. Perhaps with some surprise, as we have seen in this journey, entropy actually ended up occupying a place in all these stages.

It has found a space on the cosmic stage, containing, in fact from the beginning, an observation that had a universal flavour: *all* reversible machines extract from the environment the same amount of entropy as they dump into it, appearing all equal from the perspective of entropy, regardless of their macroscopic and microscopic make-up. This general principle suggested entropy as being a “constant of motion” for processes carried out by reversible machines. It quickly turned out that it was more interesting to look at those, much more common, processes that do *not* conserve entropy and actually use the latter to define and measure such irreversible processes. Having realised that no specific scale was attached to these reasonings, nothing prevented their application to larger and larger “machines”, up to the ultimate “machine”, the universe itself. Entropy’s elusiveness notwithstanding, in this way, the concept ascribed a first quantitative and general character to the ubiquitous dissipation, irreversibility and the second law of thermodynamics.

These developments motivated the hunt for the ingredient that rendered the neat and reversible microscopic mechanical processes to become irreversible, choosing a preferential direction in time, or at least appear to do so to the macroscopic observer. As entropy was the only quantity measuring such processes, the only way to find a *cause* for the latter was to turn to the former and explain it mechanically. This problem was a core thread in Boltzmann’s scientific life. The answer that he strove to find would eventually not be located in the realm of mechanics, but in that of probability. As we have seen, looking at entropy from this angle crucially endowed it with a microscopic interpretation. It is not a regular, but an unusual one: a *statistical* microscopic interpretation, based on weighing sets of microstates with respect to one another, rather than on any intrinsic difference between them.

This very answer, as much as it represented a crucial shift and provided clarity, was also enigmatic, and contained the pass to yet another redefinition of itself: if entropy is probabilistic, this characteristic might not be an intrinsic property of the system, but instead might be dictated by our lack of information on it, as is the case for all the classical phenomena we decide to treat probabilistically. In this way, entropy ties in with general reflections on the nature of probability and information, eventually incorporating them as integral parts. This allowed the recognition of some general properties in the function entropy that render it universally applicable in yet further contexts than those relating to thermodynamics. Interpreted in these terms, entropy branched out from the physics—even from the physics of information—to become a function of theoretic information and of which the physical cases are but applications.

Regardless of whether we now judge the various appropriations as legitimate, this chain of complementary but also idiosyncratic retellings shows the reasons behind the peculiar fruitfulness of the concept of entropy: on the one hand, each previous version was perceived by the pioneers as not satisfactory or fundamental enough and thus stimulated further search for its “truer” nature; and, on the other hand, in the fact that each new version generated a new conceptual paradigm, with its own related analogues and extensions, before eventually showing its limitations. In this process, the connections that entropy was perceived to have with irreversibility, time, the fate of the cosmos, and the nature of probability, have induced to constant speculation, and contributed to render the concept ever more alluring and open to cross-contextual borrowings, within and out of science.

By now, the reason for the title should be clear: as with Queneau’s story in *Exercices de style* [53], entropy was retold and reinterpreted in manifold ways. These were all different stories, and yet the same story in that they shared a common core. Considering its conceptual developments as a whole, entropy suggests us that perhaps the essential quality of a fruitful and thought-provoking concept is, at least in the eyes of some questioners, to call for ever more fundamental definitions, to stimulate reinterpretations, to allow for some ambiguity and plasticity to remain rather than dispel them entirely, and yet to give us one core to refer to and use as a base for our retellings.
